# Preprosthesis fracture of femoral and loosening of the femoral prosthesis during closed reduction of hip dislocation: A case report

**DOI:** 10.1016/j.ijscr.2020.06.071

**Published:** 2020-07-11

**Authors:** Jing Zeng, Qingye Qiu, Haifeng Lan, Zhiguo Wang

**Affiliations:** a Department of Orthopaedic Surgery, Liwan Central Hospital of Guangzhou, Guangzhou, 510000 China; b Department of Orthopaedic Surgery,The Third Affiliated Hospital of Guangzhou Medical University, Guangzhou, 510150 China

**Keywords:** Bipolar hemiarthroplasty, Dislocation, Prosthesis, Fracture, Loosening

## Abstract

**Introduction:**

Dislocation after bipolar hip hemiarthroplasty is an uncommon complication. Although closed manoeuvre reduction under general anaesthesia is a common and effective method for dislocation of the hemiarthroplasty. However, closed reduction leads to preprosthesis fracture of femoral and loosening of femoral prosthesis is a rare situation, which requires incision reduction and a revision hemiarthroplasty.

**Presentation of case:**

A 80-year-old female had cementless bipolar hip hemiarthroplasty due to a right femoral neck fracture. At the 11 days of postoperation, the patient had a dislocation of the hemiarthroplasty when squatting. The patient had preprosthesis fracture and loosening of the prosthesis during closed reduction. Eventually, the patient had an open reduction and femoral stem revision. There was no re-dislocation and no re-fracture at one-year follow-up.

**Discussion:**

Closed manoeuvre reduction is a common and effective method for dislocation of the hemiarthroplasty. But if not done properly, it may lead to preprosthesis fracture of femoral and loosening of the femoral prosthesis. Preprosthesis fracture of femoral and loosening of femoral prosthesis is a complex complication. This condition requires operative treatment. A personalized surgical plan and proper techniques should be done before the operation.

**Conclusion:**

Closed reduction should be performed gently to avoid preprosthesis fracture and loosening of the prosthesis. If this happens, a high-resolution CT examination should be performed immediately to evaluate the fracture and the rotation of the prosthesis. In the case of dislocation after bipolar hip hemiarthroplasty in patients with Alzheimer's disease, we hypothesize that early wearing braces to limit squat might help prevent this condition.

## Highlights

• This is the first reported case of a periprosthetic fracture of femoral and loosening of femoral prosthesis caused by closed reduction.
• Closed reduction leads to preprosthesis fracture of femoral and loosening of femoral prosthesis is a complex complication that requires operative treatment.
• Closed reduction should be performed gently to avoid this condition. If this happens, a high-resolution CT examination should be performed immediately to evaluate the fracture and the rotation of the prosthesis.
• In the case of dislocation after bipolar hip hemiarthroplasty in patients with Alzheimer's disease, we hypothesize that early wearing hip joint fixation to limit squat might help prevent this condition.

## 1 Introduction

Bipolar hip hemiarthroplasty for subcapital neck of femur fractures is a commonly performed procedure [1]. Dislocation after bipolar hip hemiarthroplasty is one of the serious complications, which not only combines the problem of difficult reduction but also may lead to increased mortality and morbidity in patients [2]. According to the literature, the incidence of dislocation of bipolar hip hemiarthroplasty using a posterior later surgical approach was between 3.8% and 10.7% [3]. Closed reduction leads to preprosthesis fracture of femoral and loosening of femoral prosthesis is a rare situation. This work is reported accord to the SCARE criteria [4].

## 2 Presentation of case

**Fig. 1:**
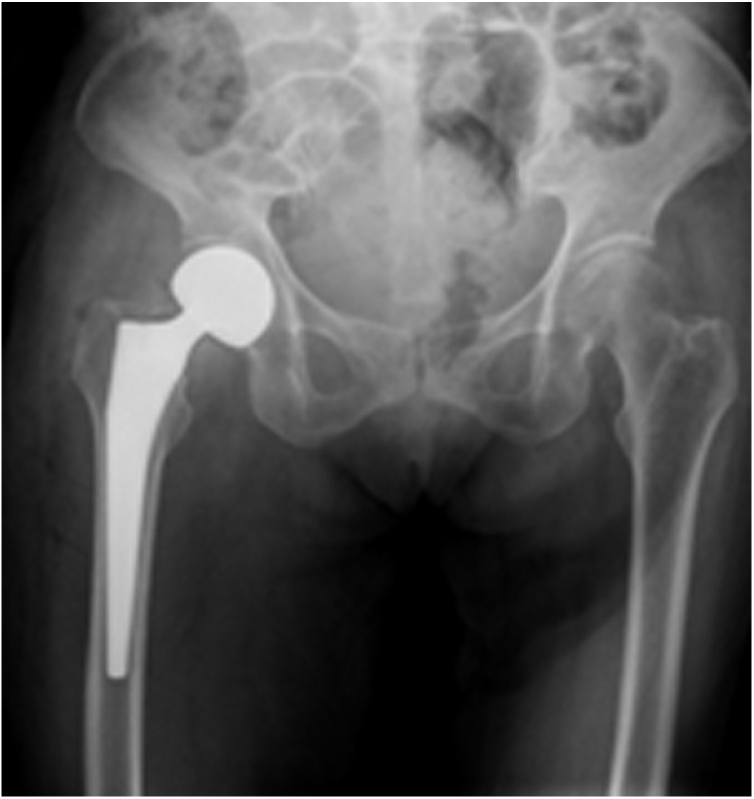
Anteroposterior radiograph of the right hip demonstrating a satisfactory hip hemiarthroplasty subsequent to a subcapital neck of femur fracture.

**Fig. 2:**
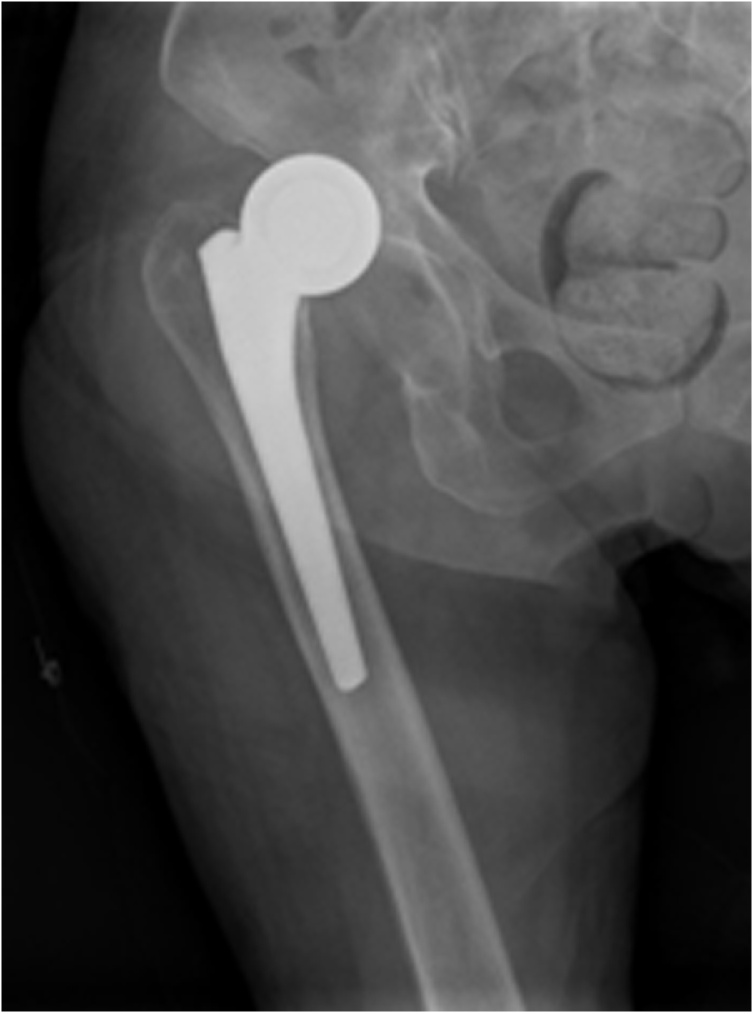
Anteroposterior radiograph of the right hipdemonstrating a dislocation of right hip hemiarthroplasty.

An 80-year-old woman with limited mobility was sent to the emergency department by a lathe with right hip pain after squatting while using the toilet. The patient was admitted to the hospital 14 days ago due to a right femoral neck fracture (Garden type III). We performed her a bipolar hip hemiarthroplast using a posterior later surgical approach (Fig. 1). She could walk with the help of a walker after surgery. She was discharged 9 days after surgery. The Harris score was 90. The patient was accompanied by mild Alzheimer's disease and no history of the neuromuscular system. She did not listen to the doctor's advice and went to the bathroom to squat the toilet 11 days following her hemiarthroplasty causing significant pain and an inability to weight bear on the right side. On examination of the patient, it was noted that the right lower limb was shortened and externally rotated, raising concern for a dislocation. A radiograph of the pelvis showed a dislocation of the hemiarthroplasty with proximal migration (Fig. 2). We judged that the poor posture of hip flexion and adduction during squatting led to the occurrence of posterior hip dislocation.

**Fig. 3:**
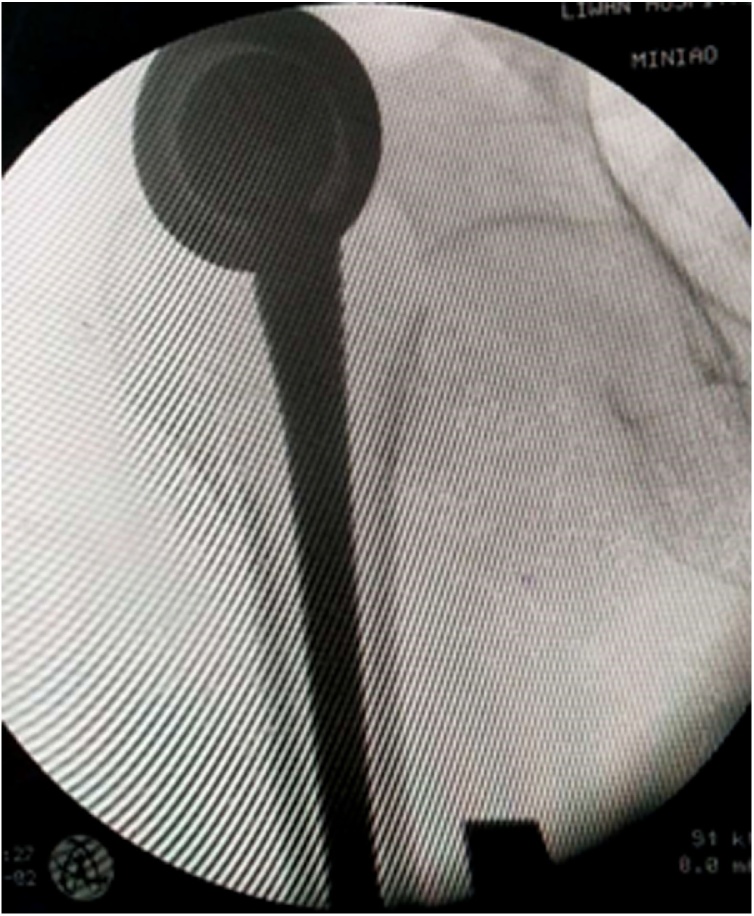
Intraoperative radiographs demonstrated dislocation still exists and preprosthesis fracture of femoral.

**Fig. 4:**
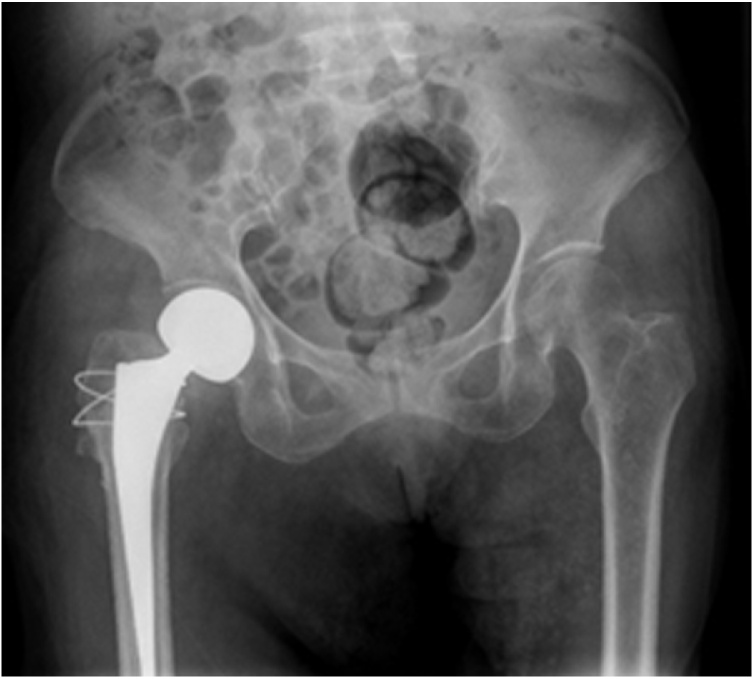
Postoperative radiograph demonstrating the revision hip hemiarthroplasty.

The next day the patient underwent a closed reduction under general anaesthesia. Unfortunately, we heard a clicking sound during the closed reduction. Radiographs demonstrated dislocation still exists and preprosthesis fracture of femoral(Vancouver classification AG). Therefore, the patient had to undergo open surgery again. Open reduction was performed through the old surgical scarusing the posterior approach. Intraoperative assessment discovered dislocation, loose rotation of femoral stem, greater and lesser trochanter fracture and displacement. Considering the poor general condition of the patient with the preoperative haemoglobin is only 79 g/L, we used a tension band to bundle the greater and lesser trochanter for resetting and fixing, and took out the original bio-type prosthesis handle and replaced it with a bone cement prosthesis handle (Fig. 4). The patient was discharged from the hospital with a hip brace and a Harris hip score of 81.There was no re-dislocation and no re-fracture at one-year follow-up (Fig. 3).

## 3 Discussion

Bipolar hip hemiarthroplasty is currently the most commonly used surgical method for the treatment of femoral neck fractures. It can effectively alleviate the pain of patients and help them restore their ability to move early. It also has the advantages of short operation time and less intraoperative bleeding [5]. However, bipolar hip hemiarthroplasty also has some potential complications, such as dislocation, loosening of the prosthesis, preprosthesis fracture, acetabular cartilage wear, and infection [6].Dislocation is one of the serious complications after bipolar hip hemiarthroplasty. Not only is it difficult to reduce the problem, but it may also lead to increased mortality and morbidity [2]. According to the literature, the dislocation rate after posterior lateral approach for bipolar hip hemiarthroplasty is 3.8%–10.7%. Most dislocations occur mainly 6 months after surgery [3]. The cause of dislocation is not clear, but it can be divided into patient factors, surgical factors and morphological factors. The patient suffered from Alzheimer's disease and did not listen to dissuasion at home, squatting on his own, resulting in hip dislocation. Therefore, in addition to strong postoperative education for patients with dementia, we recommend that they should wear braces at home to limit their squat to reduce the risk of dislocation.

Closed reduction is the most commonly used method for dislocation of the hip joint. Most dislocations can be successfully reset. Very few patients require traction reduction or open reduction. Although closed reduction has a high success rate, if it is not performed properly, complications such as preprosthesis fracture, loosening of the prosthesis, acetabular fracture, and dissociation are prone to occur. Therefore, routine CT examinations before surgery are necessary to evaluate whether the prosthesis is loose and whether there is a fracture around the prosthesis.

Preprosthesis fracture of femoral and rotation of the prosthesis during closed reduction are caused by iatrogenic factors. It may be related to the abduction and distal rotation of the femur in the case of insufficient stretching during the operation. At present, there are no reports about the incidence of periprosthetic fractures after bipolar hip hemiarthroplasty, but according to the literature, the incidence of periprosthetic fractures after total hip arthroplasties is 0.4–3.5% [7]. Most of these fractures are caused by low-energy falls, and most of them are accompanied by loosening of the femoral prosthesis [8]. Patients with preprosthesis fracture of femoral frequently require surgery to restore mobility. This not only increased the patient's pain and financial burden, but also the failure rate of the second operation was higher, reaching 16.5% [9]. As a result, the 1-year mortality rate of patients increased by 11–13.2% [10]. The most commonly used classification system is the Vancouver classification [11]. In this report, the patient's fracture classification was type A (AG + AL), with fracture displacement, and an open reduction and internal fixation should be performed. During surgery, we use tension bands to restore greater and lesser trochanter. Due to the rotation of the prosthesis, we further removed the original femoral prosthesis and replaced it with a cemented prosthesis to increase the stability of the prosthesis.

## 4 Conclusion

To our best knowledge, This is the first reported case of a periprosthetic fracture of femoral and loosening of femoral prosthesis caused by closed reduction. Closed reduction leads to preprosthesis fracture of femoral and loosening of femoral prosthesis is a complex complication that requires operative treatment. Closed reduction should be performed gently to avoid this condition. If this happens, a high-resolution CT examination should be performed immediately to evaluate the fracture and the rotation of the prosthesis. In the case of dislocation after bipolar hip hemiarthroplasty in patients with Alzheimer's disease, we hypothesize that early wearing hip joint fixation to limit squat might help prevent this condition.

## Conflicts of interest

The authors declare that they have no conflict of interest.

## Funding

There are no sources of funding.

## Ethical approval

Not required for case reports at our hospital. Single case reportsare exempt from ethical approval in our institution.

## Consent

Written informed consent was obtained from the patient forpublication of this case report and accompanying images. A copyof the written consent is available for review by the Editor-in-Chiefof this journal on request.

## Author contribution

Jing Zeng: managed the patient and did the surgery, design of the study, data interpretation and analysis, revision.

Qingye Qiu: patient care, revision, corresponding author.

Haifeng Lan: data collection, revising critically,wrote the manuscript.

Dongdong Wu: review and editing, data analysis,wrote the manuscript.

All authors read and approved the final manuscript.

## Registration of research studies

NA.

## Guarantor

Dr. Jing Zeng.

## Provenance and peer review

Not commissioned, externally peer-reviewed.
